# Endemic foot and mouth disease: pastoral in-herd disease dynamics in sub-Saharan Africa

**DOI:** 10.1038/s41598-019-53658-5

**Published:** 2019-11-22

**Authors:** I. McLachlan, G. Marion, I. J. McKendrick, T. Porphyre, I. G. Handel, B. M. deC. Bronsvoort

**Affiliations:** 1 The Epidemiology Economics and Risk Assessment (EERA) Group, The Roslin Institute and Royal (Dick) School of Veterinary Studies, University of Edinburgh, Easter Bush, Edinburgh, Midlothian, Scotland United Kingdom; 2 Biomathematics and Statistics Scotland, Edinburgh, United Kingdom; 3 Royal (Dick) School of Veterinary Studies, University of Edinburgh, Easter Bush, Edinburgh, Midlothian, Scotland United Kingdom

**Keywords:** Mathematics and computing, Infectious diseases, Infectious diseases, Mathematics and computing

## Abstract

Foot and mouth disease (FMD) burden disproportionally affects Africa where it is considered endemic. Smallholder livestock keepers experience significant losses due to disease, but the dynamics and mechanisms underlying persistence at the herd-level and beyond remain poorly understood. We address this knowledge gap using stochastic, compartmental modelling to explore FMD virus (FMDV) persistence, outbreak dynamics and disease burden in individual cattle herds within an endemic setting. Our analysis suggests repeated introduction of virus from outside the herd is required for long-term viral persistence, irrespective of carrier presence. Risk of new disease exposures resulting in significant secondary outbreaks is reduced by the presence of immune individuals giving rise to a period of reduced risk, the predicted duration of which suggests that multiple strains of FMDV are responsible for observed yearly herd-level outbreaks. Our analysis suggests management of population turnover could potentially reduce disease burden and deliberate infection of cattle, practiced by local livestock keepers in parts of Africa, has little effect on the duration of the reduced risk period but increases disease burden. This work suggests that FMD control should be implemented beyond individual herds but, in the interim, herd management may be used to reduced FMD impact to livestock keepers.

## Introduction

Many livestock keepers in sub-Saharan Africa (SSA) experience annual outbreaks of foot and mouth disease (FMD)^1^. Losses^2–4^, mainly as a result of reduced milk production and loss of draft power^5^, are significant and pastoral and smallholder farmers are vulnerable due to their reliance on livestock for their livelihoods and food security^6^. Although formal economic losses are not typically the primary concern of smallholders^5^ and pastoral livestock keepers in low and middle income (LMICs) settings, reduced income from livestock also limits spending capability on human health^7^, education and food. Livestock also remain important for social status and cultural identity in large parts of SSA^8^. Therefore further efforts need to be made to understand and control FMD in endemic settings.

South America has made good progress in reducing FMD incidence through vaccination and outbreak response surveillance^9^. However, it is not clear that such successes will be easy to replicate in remaining endemic regions. Vaccination coverage in South America, where huge control efforts have been made, is estimated to be 146.1%^10^, reflecting vaccination of animals more than once per year on average. In contrast it is estimated that 5.5% of the African cattle population vaccinated^10^. Resources and spending on FMD control varies widely between endemic countries^11^. The lack of resources available for animal disease control in SSA means large-scale government control programs are likely to be limited and therefore the burden of control falls on individual livestock keepers and their communities^12^. Control measures implemented vary between smallholders. Although a study of smallholders in Kenya found vaccination and movement restriction to be the most common control measures, nearly a third of those surveyed did nothing to prevent disease^13^. Whilst just under half of respondents vaccinated, less than a quarter had vaccinated within the last 6 months – the recommended vaccination frequency for FMD. Willingness to pay for vaccination is likely to be greater when the risk of disease is immediate (i.e. emergency vaccination) but poor access to veterinary services and a lack of resources to purchase drugs inhibit the implementation of vaccination based control among smallholders/pastoralists in SSA^14^. Even limited improvements to current control in SSA are predicted to have a socioeconomic benefit for livestock keepers^5^. Therefore, in the near to medium term, the best strategy for control is likely to involve targeting individual livestock keepers, which requires improved understanding of within-herd dynamics. It is important that those undertaking control perceive it to be successful and cost effective. This is particularly true in the context of low-risk, low-reward farming systems where future planning for disease control is limited and there are often significant barriers to investing in increased productivity^11^. In addition, long term participation and compliance are important for sustained disease control. Therefore, before encouraging livestock keepers to invest in control it is important to understand how they will benefit as individuals.

Identification of effective control measures for FMD in pastoral West and Central Africa requires an understanding of endemic FMD that we currently do not have^11^. There are seven distinct serotypes of FMD virus (FMDV) (type A, O, C, Asia1 and Southern African Territories (SATs) 1, 2 and 3)), which have different geographical distributions – all but Asia1 have been found in SSA^15^. There is little evidence for cross protection between the different serotypes, therefore, they can be explored largely independently. However, different serotypes of FMD virus are clinically indistinguishable and multiple serotypes co-circulate in endemic regions in SSA. This complicates efforts to distinguish the dynamics of the individual serotypes from the observed disease in the field. Understanding of FMD in endemic regions is largely informed by cross-sectional studies. While this is beneficial for providing an overview of FMD it can be difficult to understand ongoing disease dynamics, particularly at the herd-level.

The potential role of carriers in FMD dynamics and persistence is an example of an open question where the scientific debate has yet to arrive at a consensus. A carrier is, by definition, an individual from which virus can be recovered more than 28 days after infection^16^. It is unclear whether carriers contribute to transmission of FMD. There are anecdotal reports of transmission^17^ but until recently this had not been shown in experimental studies^18^. Arzt *et al*. have shown transmission of FMD from carriers to naïve cattle through transfer of oropharyngeal fluids^19^. Although not reflective of transmission in field settings, this finding once again brings into question the role of carriers in FMD dynamics. Although studies have explored the epidemiology of carriers due to the lack of evidence for transmission it is assumed that the role of carriers in disease dynamics within the herd is negligible. It is important to understand if this is true and whether the presence of carriers would alter advice given to herders, particularly as vaccination does not protect against the development of carrier status^20^.

Modelling provides a valuable tool for bringing together existing knowledge within a coherent and logical framework, and highlights areas where more, better quality data are required to improve our understanding of the epidemiology of a disease. However, most FMD modelling focuses on FMD-free settings where availability of good quality data has enabled detailed study on the mechanisms underlying the spread of FMDV and improved its control in epidemic settings^21–23^. Furthermore, varying levels of immunity and greater movement and interaction between herds are generally not relevant in FMD-free settings but need to be considered in endemic settings. The few endemic FMD^24,25^ models used in attempts to understand persistence within endemic countries and to estimate certain parameters^26^ have not explored the within-herd dynamics of FMD in cattle in an endemic setting. Disease dynamics vary across different scales so in order to understand within herd dynamics disease transmission needs to be modelled at such a scale. Here we develop a stochastic compartmental model for within-herd FMD dynamics and parameterise it using the best available information from the literature on FMD in smallholder pastoral cattle herds in endemic settings. We use this framework to enhance understanding of endemic FMD by addressing the following questions: (1) How does the role of carriers and infection memory affect the long-term persistence of FMDV in cattle herds in a setting typical of pastoralists in SSA?; and (2) How does herd management affect disease recurrence and burden?

## Results

### Persistence, carriers and infection memory

**Figure 1:**
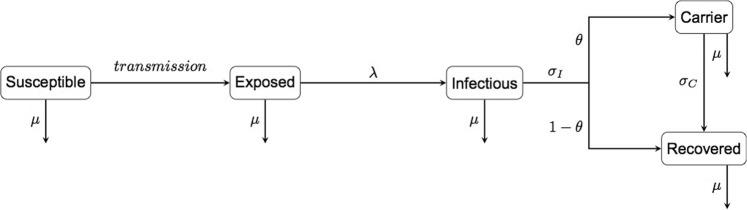
Within-herd FMD model. β - transmission parameter, λ - per-capita rate of onset of infectiousness, σ_I_ - per-capita rate of recovery from the infectious period, θ - proportion of infectious individuals that become carriers, σ_C_ - per-capita rate of recovery from the carrier state and μ - per-capita rate of removal from the herd.

We developed a stochastic, compartmental model to explore disease persistence and dynamics. The model structure is of the form susceptible-exposed-infectious-recovered-carrier (SEIRC) (Fig. 1). By varying the proportion of infectious individuals that become carriers before recovering (θ) we explored scenarios with and without carriers. We modelled a herd of 70^27^ individuals. We assumed no disease associated death in our model as FMD related mortality is very low in adult cattle^28–30^ and individuals leaving the population were replaced with susceptible individuals. A basic reproductive number (R_0_) of 4^24,31–35^, latent period (T_latent_) of 4.6 days^36,37^ and the infectious period (T_infectious_) 1.7 days^36^ were used. To maintain a consistent R_0_, reflective of observed outbreaks, the transmission parameter (β) was adjusted for each of different scenarios (see Methods). The model was initialised with a single infectious individual and evaluated through 50 years the level of prevalence in the herd while accounting for herd management practices. Once simulations ended, outbreaks were classified as ‘small’ or ‘large’ based on the total number of incident animals that were infected within the herd during the study period. Here, large outbreaks are defined as ones in which more than 10% of the population are infected (all other outbreaks are classified as small). The results presented represent 10000 simulations of the model.

Using this model, we explored the herd-level persistence and dynamics with and without a carrier state (θ = 0.5^38,39^ and θ = 0). To explore whether disease can persist within a single herd we simulated outbreaks and recorded size and duration. Outbreak duration was measured as the time during which exposed or infectious individuals were present in the herd. Estimates of carrier characteristics from empirical studies vary and it is unclear if such individuals can transmit virus. To investigate whether a carrier state may alter persistence and disease dynamics we have used a range of relative (i.e. relative to that from the infectious state) transmission rates (ω). Viral persistence was measured as the time during which exposed, infectious or carrier individuals were present in the herd and the number of infectious individuals during this time was recorded. The reduced risk period (T_ReducedRisk_) is defined as the time interval, following the initial outbreak, for which the effective reproductive number (R_t_) is less than 1. We measured the proportion of outbreak, initiated by a single carrier, that were large. Through model simulation we also investigated the relationship between the inter-outbreak period and the size of the subsequent outbreak resulting from disease incursion into the herd.

**Figure 2:**
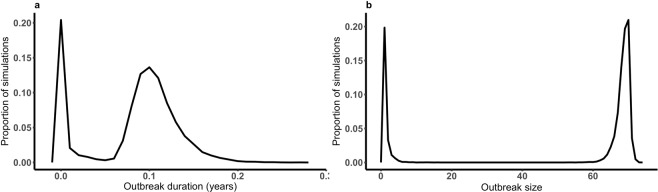
Disease cannot persist within a single herd. The mean duration of large outbreaks is 0.083 years (0.99 months) with 100% of outbreaks lasting less than 0.27 years (3.2 months) (**a**). The majority of outbreaks are large although some small outbreaks are observed (**b**).

In the scenario in which no carrier state is involved in the dynamics of FMDV in a typical cattle herd disease cannot persist – no simulations showed FMDV remaining in the herd within the 50 years simulated. Indeed, the mean duration of all simulated outbreaks was 0.083 years (95% CI: 0.082 to 0.084 years) and the maximum less than 0.25 years (Fig. 2a). Between 1 and 7 individuals were infected in 25% of the simulations whereas 75% of simulations permitted outbreaks of 61 to 72 infected individuals (Fig. 2b), for a herd size of 70 animals. Although the herd size was fixed to 70 individual cattle, 4% of simulations showed a number of incident cases that were greater than 70, highlighting that replacement individuals will ultimately be infected if introduced into a herd in which FMD is actively circulating.

**Figure 3:**
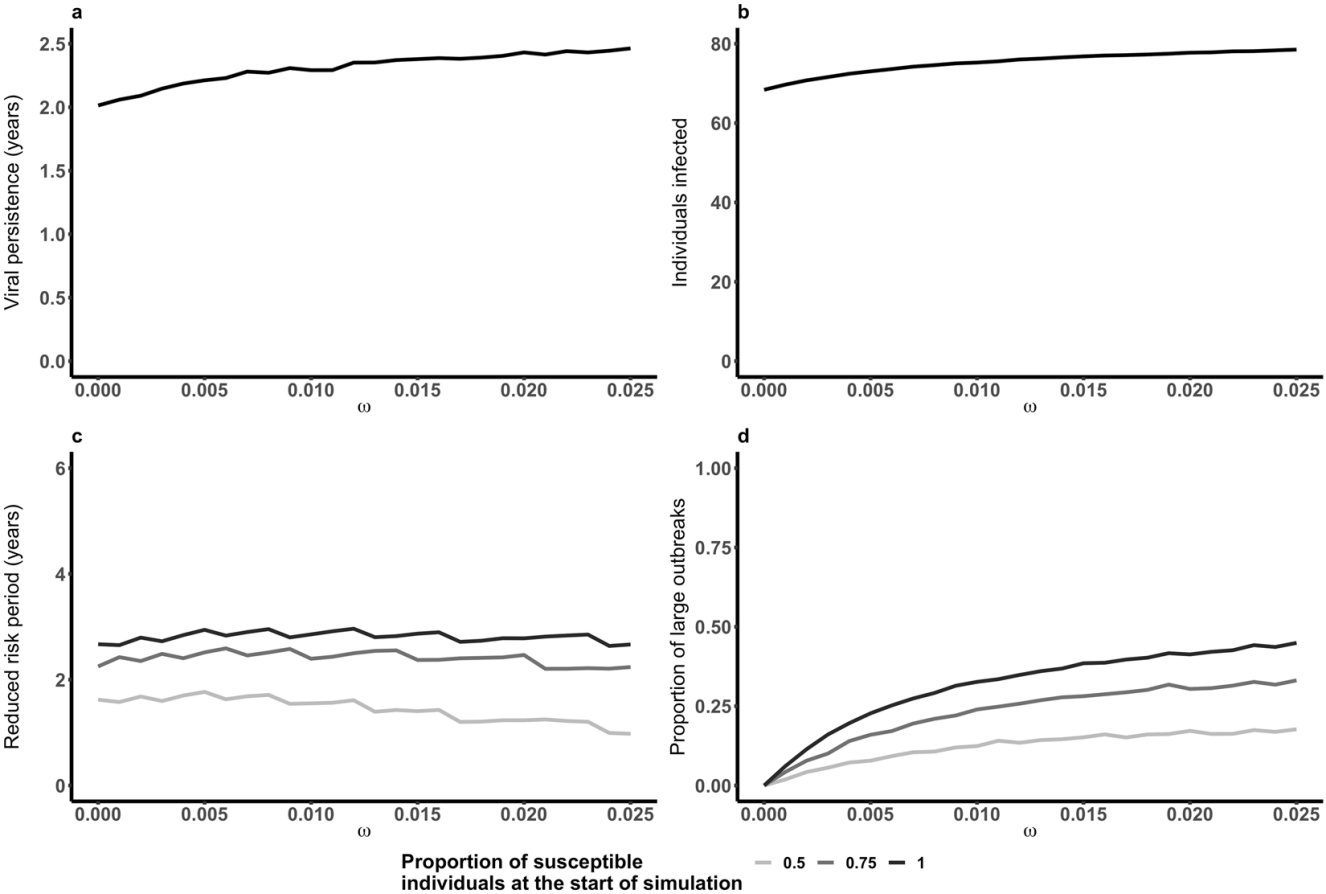
Impact of Carriers. Carriers increase viral persistence within a herd following an initial disease incursion regardless of the relative transmission from carriers (ω) (**a**). The total number of individuals infected whilst virus persists is similar to a herd without carriers (**b**). The average reduced risk period increases with a very small increase in ω but then decreases as ω increases further (**c**). When carriers are able to transmit FMDV there is a (non-zero) probability that a single carrier initiates a large outbreak (**d**).

In contrast, when adding a carrier state, the mean viral persistence within the herd is markedly prolonged, with a mean persistence increasing from less than 0.25 years to more than 2 years. In addition, increasing the value of ω from 0 to 0.025 yields only minor changes (Fig. 3a). However, mean disease persistence (outbreak duration) remains similar to the persistence seen in models without carriers, although this again increases slightly as ω increases (Fig. S1a, Supplementary Material). The mean total number of individuals becoming infectious whilst virus persists does increase as ω increases, but the difference is less than 10 for the range of ω explored (Fig. 3b). Mean T_ReducedRisk_ decreases only slightly as ω increases (Fig. 3c). When ω is high large outbreaks are, on average, smaller (Fig. S1b, Supplementary Material) and therefore less time is required for the herd to regain a level of susceptibility that would support further outbreaks. The risk of a single carrier initiating a large outbreak is shown in Fig. 3d. Increased ω increases the probability that a single carrier will initiate a large outbreak, however, the probability of a large outbreak is lower than if the initial individual were infectious. Overall these results suggest that, other than the duration of viral persistence, measures of herd-level disease dynamics are similar whether carriers are included in the model or not. Therefore, results presented in the remainder of the paper use the simpler model without carriers (however, for completeness additional results with carriers are shown in the Supplementary Material; Figs S1–S4).

**Figure 4:**
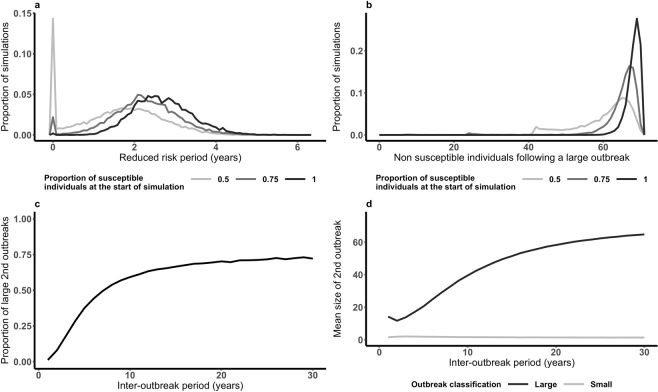
Memory of herd immunity following a large outbreak. Following an outbreak there is a period where the herd is at reduced risk (T_ReducedRisk_) of further outbreaks (**a**). Higher immunity (more non susceptible individuals) within the herd following a large outbreak (increased by greater susceptibility prior to the outbreak) extends T_ReducedRisk_ (**b**). The longer the inter-outbreak period the greater the probability that the subsequent outbreak will be large (**c**) and these large outbreaks will, on average, infect more individuals (**d**).

Herds have memory of pervious outbreaks as currently immune individuals exposed in previous outbreaks cannot be infected. Herd memory does affect future outbreaks (Fig. 4). Following large outbreaks there is a period where the herd is at reduced risk of further outbreaks (Fig. 4a). The mean T_ReducedRisk_ is longer when susceptibility prior to the initial outbreak is high (1.61 years when 50% of the herd is susceptible and 2.68 years when 100% of the herd is susceptible). Generally, higher immunity following an outbreak results in longer T_ReducedRisk_, and higher susceptibility prior to an outbreak results in higher average immunity (Fig. 4b) following a large outbreak. The longer the inter-outbreak interval the higher the probability that subsequent outbreaks will be large (Fig. 4c). Given that a large outbreak has occurred, when the inter-outbreak period is 7 years there is a greater than 50% probability that the second outbreak will also be large. When the inter-outbreak period is greater than 20 years the probability that the second outbreak is large is similar to the probability of a large outbreak in a fully susceptible population. As the inter-outbreak interval increases so too does the average size of the second outbreak – the average size of large outbreaks increases whilst the average size of small outbreaks remain similar (Fig. 4d). The average size of small outbreaks changes very little regardless of the inter-outbreak interval.

### Implication of herd management on outbreak frequency and burden

We explored two herd management practices to understand how they affect the T_ReducedRisk_ following a large outbreak: changing average T_in-herd_ by varying the population turn-over (μ); and the efficacy of the practice of deliberate infection. The former is largely under the control of herders as it reflects the replacement rate of animals within the herd as well as their typical lifespan. Deliberate infection purposely exposes animals to infectious material when disease is nearby. It is still practiced as a methods of FMD control in some developing countries to reduce uncertainty surrounding infection and the duration of disease impact^40^ and to provide immunity to animals that survive infection. In our modelled scenario when an individual other than the initially infected individual became infectious a proportion of the remaining susceptible population was deliberately infected (i.e. was made to enter the exposed state). FMD burden associated is high and the impact is felt not only during the outbreak but afterwards as recovered individuals may be less productive. We explored whether average T_in-herd_ may be used to minimise disease burden associated with FMD (without preferentially removing animals of particular disease states).

Burden was measured separately for infectious and recovered individuals as the total number of days per year spent in each of the disease states. Overall burden combines these two measures, weighting a day in the recovered class as a fraction (δ) of a day in the infectious class (i.e. we have chosen to weight infectious burden more highly than recovered burden). A study of a dairy farm in Kenya reported reduction in daily milk yield during an outbreak, following the outbreak milk yield recovered but not to pre-outbreak levels^41^. The robustness of optimal strategies to reduce burden was assessed using a range of such weightings.

**Figure 5:**
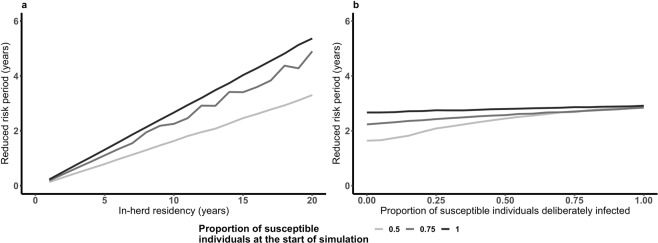
Impact of herd management on the reduced risk period (TR_educedRisk_) following a large outbreak. Shorter average in-herd residency shortens T_ReducedRisk_ (**a** – the rate of population turn-over (μ) is varied), increasing the proportion of individuals deliberately infected results in a longer mean T_ReducedRisk_ following an outbreak (**b** – once an outbreak is detected deliberate infection of a proportion of the remaining susceptible individuals occurs).

Results show that herd management practices can alter the T_ReducedRisk_ following a large outbreak (Fig. 5). T_in-herd_ has a strong impact on the T_ReducedRisk_ (Fig. 5a). Shorter T_in-herd_ results in a shorter mean T_ReducedRisk_. Our model assumes that individuals leaving the herd are replaced with susceptible individuals. When more of the replacement individuals are in the recovered class rather than susceptible the mean T_ReducedRisk_ is longer (Fig. S6, Supplementary Material). When the population is partially susceptible and a higher proportion of the susceptible population is deliberately infected there is a slight increase in the average T_ReducedRisk_ (Fig. 5b). This is because deliberate infection increases the average outbreak size.

In fully susceptible populations, 100% effective deliberate infection increases T_ReducedRisk_ by 0.24 years. Increasing the in-herd residency from 10 to 11 years would have a similar impact (increasing T_ReducedRisk_ by 0.27 years). When the herd is 75% susceptible prior to the outbreak increasing in-herd residency from 10 to 12 years increases T_ReducedRisk_ equivalent to deliberate infection. In herds where 50% of the herd is susceptible prior to the outbreak increasing in-herd residency from 10 to 18 years is required to produce the same increase in T_ReducedRisk_ as deliberate infection.

**Figure 6:**
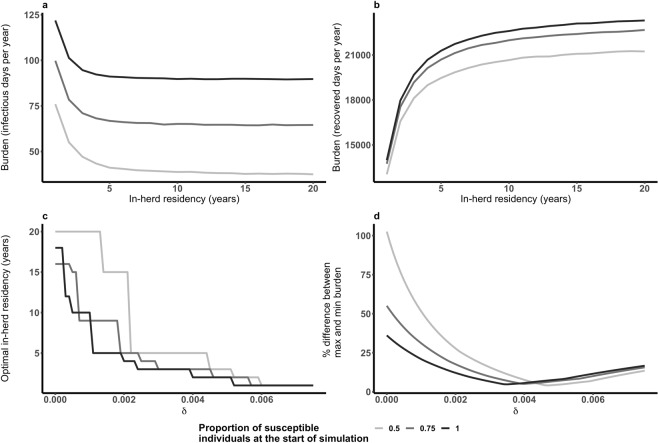
Disease burden varies with average in-herd residency. Mean infectious (I) disease burden decreases with increasing average in-herd residency (**a**). Mean recovered (R) disease burden increases with increasing average in-herd residency (**b**). Higher relative contribution of recovered individuals (δ) to overall disease burden favours shorter in-herd residency (**c**). There is greater variation between maximum burden relative to minimum burden when δ is low (**d**).

Mean infectious disease burden decreases as average T_in-herd_ increases (Fig. 6a) whilst the mean recovered disease burden increases (Fig. 6b). Longer T_in-herd_ is the optimal strategy when recovered burden does not contribute to overall burden (Fig. 6c). A higher δ favours shorter T_in-herd_. We have measured the variation between the minimum and maximum estimates for burden as a percentage of minimum disease burden (Fig. 6d). Across the parameter space, the percentage difference between the minimum and maximum total burden varies between 5 and 106% relative to the minimum burden. There is more variation between the minimum and maximum estimates for burden when only infectious individuals contribute to the overall burden. Increasing the proportion of the population deliberately infected increases both infectious and recovered disease burden (Fig. S5, Supplementary Material).

## Discussion

The negative impact of FMD in endemic regions has been well documented^5–7,10^ but understanding of disease dynamics is poor^8,11^. Countries in FMD endemic SSA are at the very early stages of the Progressive Control Pathway for FMD, prior to the implementation of control^12^. In such countries resources needed to control FMD compete with spending on health and education and other agricultural programs supporting staple foods^42^. Therefore national or regional level control programmes are likely to have limited impact in the short to medium term and individuals must continue to manage their herds as best they can in the context of FMDV circulation. Our study aims to support such management systems. Specifically we address a current gap in the literature by developing a within-herd transmission model based on the best available information on FMD in endemic regions, contributing to improved understanding of within herd disease dynamics. Our analysis of this model addresses a number of important issues and highlights important gaps in FMD knowledge and associated herd management practices in endemic regions. This analysis suggests that FMD cannot be sustained in an isolated herd. Hence, repeated introduction of virus from outside the herd is likely responsible for the frequent outbreaks observed in endemic regions^43,44^. Our study has also highlighted the limited availability of information about smallholder farmers and pastoralists and how they are affected by FMD, in particular a lack of longitudinal data. Availability of good quality data will be important in identifying the difference between outbreaks observed by smallholders and the true underlying disease dynamics.

Smallholder and pastoralist livestock keepers in endemic regions are caught in a cycle of repeated outbreaks. To obtain insight into how this ongoing threat of outbreaks affects disease dynamics within individual herds, we considered pairs of outbreaks; a large initial or first outbreak, against a background of varying levels of herd susceptibility reflecting the range of within-herd in endemic regions^45^, followed by a second or subsequent outbreak caused by disease incursion. We refer to the time between the end of the first outbreak and the subsequent incursion as the inter-outbreak period. Large secondary outbreaks, where more than 10% of individuals are infected, are rare when incursions occur soon after the end of the initial outbreak. In contrast, larger secondary outbreaks are more likely when the inter-outbreak period is longer or when immunity prior to the initial outbreak is higher. However, the herd susceptibility prior to the initial outbreak exerts a strong influence on whether a second outbreak, one year later, is large. When pre-initial outbreak susceptibility is only 50% the probability of a large second outbreak one year after the first is only 0.12. Full herd susceptibility prior to the first outbreak reduces this probability to 0.02. To understand this difference consider that the typical initial outbreak size and levels of immunity following it are reduced when herd susceptibility is lower. Therefore the herd will typically recover susceptibility to be able to support another outbreak more quickly.

The inter-outbreak period also has a strong effect on the characteristics of a second outbreak. The probability that a large outbreak follows the initial large outbreak increases above 50% when the inter-outbreak period is greater than 7 years. A small sample of within-herd outbreaks in Cameroon suggest that outbreaks infect around 37% of the herd, with outbreak size varying between 15 and 71%^46^. These outbreaks are by our definition large and on average leave 63% of the herd susceptible. If typical of the near annual outbreaks observed within pastoral herds in endemic regions^43,44^ this suggests that consecutive outbreaks are caused by different strains of FMDV with poor cross-immunity as it is unlikely herds will regain susceptibility to a given strain quickly enough to experience yearly outbreaks of this size. This is consistent with the suggestion from Bronsvoort *et al*. that the appearance of persistence could be achieved through different FMDV strains passing through a population^47^. This is supported by the recent observation in eastern Africa of waves of outbreaks each with a single dominant serotype^7^. Clinical signs of FMD are indistinguishable between serotypes but are typically relied upon to measure the size and impact of outbreaks in endemic settings. Thus although observationally disease may persist it is likely that the dominant strain changes. Limited data across endemic regions^26^ makes it difficult to confirm this hypothesis, but the model-based analysis presented in this paper supports the idea that observed frequency of outbreaks in endemic regions is likely due to co-circulating strains of FMDV. Although challenging to collect, longitudinal data would also enable better understanding of the dynamics of FMD and data from different geographical locations would provide information on regional differences^45,48^.

Our model-based analysis contributes to the ongoing debate around the role of carriers in FMD dynamics. We explore a range of relative transmission rates from carriers (ω), reflecting uncertainty of the contribution of carriers to FMD transmission. To enable direct comparison, an equivalent within-outbreak R_0_ value was used in all simulations, both with and without carriers. We show that the existence of a carrier state can increase mean viral persistence within an isolated herd from less than one month to more than 2 years but does not result in indefinite persistence. Crucially, the inclusion of carriers in our model does not reproduce the frequency and size of outbreaks observed in the field^43,44^. Other characteristics explored, including the effect of herd management strategies on the reduced risk period (T_ReducedRisk_) and disease burden, are similar to those in the model without carriers. Mean outbreak duration and total infection increase slightly as ω increases but are similar to results without carriers. The mean T_ReducedRisk_ remains fairly constant as ω increases; however, carriers do represent an additional source of disease risk. The probability that a carrier initiates a large outbreak is lower than for an infectious individual, but, as expected, increases as ω increases. The potential for carriers to start outbreaks is important in FMD-free settings and is a major driver for the use of culling to control outbreaks. Whether carriers are able to transmit disease or not, our results suggest that their impact on FMD dynamics in individual pastoralist herds is small. However the prolonged infectiousness of herds when carriers are present provides greater opportunity for transmission between herds and may have implications for regional disease persistence. This effect is likely to become more important as control progresses towards eradication/elimination.

We find that herd management practices have potential to influence outbreak frequency and manage burden. High levels of herd immunity reduce outbreak risk. Pastoralists tend to rely on traditional and/or reactive treatments^49,50^ and as vaccination is not a common practice in FMD endemic pastoralist Africa we assume that all immunity is naturally acquired and life-long. Our results that show longer average in-herd residency (T_in-herd_) increases mean T_ReducedRisk_. In pastoral systems most trade results in animals being removed from the herd while replacement animals tend to be bred by the livestock keepers. One study showed 93.3% of new entries into the herd resulted from births with the remaining 6.7% from purchases^51^. FMD mortality is low^10^ but negatively affects herd fertility^52^ and is likely to slow population turn-over and increase costs. Previous exposure to disease may have further implications for herd dynamics (not explored here) if livestock keepers choose to sell less productive, previously exposed, individuals. However, this has not been observed in pastoralist communities in the Far North Region of Cameroon^49^ and would more likely occur in more profit-orientated commercial farms. Here we show that it may be possible to minimise disease burden without preferentially selling animals of a particular disease state. The optimal strategy depends on a number of factors including, which disease state experiences most of the negative effects of FMD, the outbreak size, and the pathogenicity of the viral serotypes^30,53^. Because of these sources of uncertainty, and the effect of (unknown) variation in herd immunity prior to disease incursion, further work is required before making specific recommendations regarding such management of small herds.

The deliberate infection of livestock may seem counterintuitive, but it is still practised as it reduces uncertainty and variability of infection and the overall likely duration for which the herd has clinical disease^40^. The time during which the herd is immobilised due to disease is reduced allowing the search for better grazing or markets to resume more quickly (factors not considered in our analysis). Higher levels of success in inducing infection increases the mean T_ReducedRisk_ due to the occurrence of larger average outbreaks than would be predicted without such intervention. This beneficial effect on the mean T_ReducedRisk_ is small but is more apparent when the herd has reduced susceptibility prior to the outbreak, which may be more likely in endemic regions. Our results suggest that deliberate infection, to reduce short-term uncertainty surrounding infection, increases disease burden on average. Mean outbreak size increases with greater success in deliberately causing infection, thereby increasing burden associated with infectious individuals. Larger average sizes of outbreaks also increase the number of individuals who may experience negative effects resulting from FMD after the outbreaks end, increasing disease burden in the long term. These results suggest that it would be beneficial for herders to cease this practice however further comparison between the short and long-term costs and benefits would need to be conducted to provide more definitive advice. In addition to increasing the number of animals who may experience negative effects of FMD after an outbreak has ended it has also been suggested that deliberate infection of animals with FMD may increase the probability of individuals becoming carriers^42^. Our findings suggest the implications, at the herd level, of carriers resulting from deliberate infection is likely to be minimal.

## Conclusions

Our model strongly suggests that disease cannot persist without re-introduction of FMDV within a single herd. Even disease transmitting carriers are insufficient for disease persistence. Previous models have also struggled to replicate disease persistence^24^ highlighting the complexity of endemic systems and the need for additional work. We show there is a reduced average risk period following outbreaks which is typically longer than the average period between outbreaks in FMD endemic regions. Although herd management practices alter the rate at which herds regain susceptibility to disease our analysis suggests it is implausible that herds to experience yearly outbreaks of the same FMDV serotype. Neither the reduced risk period nor the effects of herd management practices are significantly affected by the addition of a carrier state. Future work would benefit from a meta-population model approach, incorporating transmission between herds and risk of transmission from wildlife to better understand between-herd persistence and disease recurrence within herds. Such modelling studies would benefit from additional endemic region data on the frequency of outbreaks and the serotypes responsible, to disentangle the observed outbreaks from the dynamics of the circulating serotypes. The majority of farming in FMD endemic regions operates under a low-risk, low-reward system and as a result particular care needs to be taken when making recommendations regarding FMD control strategies. Better informed models would increase confidence in recommendations targeted at smallholders in endemic regions.

## Methods

### Model structure

We develop a stochastic, compartmental model of the form susceptible-exposed-infectious-recovered-carrier (SEIRC) (Fig. 1) in order to investigate within-herd FMD dynamics in endemic regions. The model is Markovian; times spent in each state follow an exponential distribution with average residence time given by the reciprocal of the rate of transition out of the given state (Table 1). Susceptible (S) individuals become infected at a rate determined by frequency-dependent transmission. Upon infection, susceptible individuals enter an exposed state^36,37^ (E) but are not capable of transmitting virus. Following a latent period exposed individuals become infectious (I) at a per-capita rate λ. Individuals leave the infectious state at a per-capita rate σ_I_. A proportion (θ) of these individuals become carriers^16^ (C) and the remaining fraction (1-θ) enter the recovered state (R). Individuals leave the carrier state at per-capita rate σ_C_ entering the recovered state. Natural immunity has been shown to be long-lived in cattle^54,55^ and therefore we have assumed that those in the recovered state have life-long immunity. No disease induced mortality is considered due to the low mortality associated with FMD infection in cattle^28,29,44^. However, all disease states are subject to a per-capita rate of population turnover, μ, which represents both natural deaths and other removals from the herd. A constant population was maintained by replacing individuals leaving the herd with susceptible individuals. The model was coded in C + + using Xcode (version 8.1) and implements the Gillespie direct method^56^.

**Table 1:** Events changing the population in response to disease.

Event	Equation	Population Change
Birth/Death* (turnover)	*μS* + *μE* + *μI* + *μR* + *μC*	−1 S/E/I/R/C and +1 S
Infection	$\frac{\beta S(I+\omega C)}{N}$	−1 S and +1 E
Becoming infectious	*λE*	−1 E and +1 I
Entering a carrier state	*θσ*_*I*_*I*	−1 I and +1 C
Recovery from the infectious state	(1−*θ*)*σ*_*I*_*I*	−1 and +1 R
Recovery from the carrier state	*σ*_*c*_*C*	−1 C and +1 R

*The equations shown refer to the death rate in the population. In the model birth occurs at the same time as death to maintain a constant population size. The overall event therefore corresponds to turnover in the herd.

### Model parameterisation to reflect smallholder subsistence livestock keepers in Africa

Our model herd has 70 individuals, reflecting an estimate of average herd size of subsistence livestock keepers in Cameroon^27^. In all simulations we have used an R_0_ of 4, which is within the range of reported R_0_ in endemic regions^24,31,32,34,57^. To investigate different scenarios we have varied parameters from baseline values (Table 2). For each scenario we have calculated the transmission parameter ($\beta =\frac{{R}_{0}}{1+(\frac{\omega {\sigma }_{I}\theta }{\mu +{\sigma }_{C}})}\times \frac{(\lambda +\mu )({\sigma }_{I}+\mu )}{\lambda }$). The average duration of the latent period (T_latent_) is 4.6 days, and the average duration of the infectious period (T_infectious_) 1.7 days^36^. Background population turnover is assumed to be $\frac{1}{10}$ years^−1^. This is comparable to $\frac{1}{11.08}$ years^−1^ as used by Jemberu *et al*.^58^. Ducrotoy *et al*.^51^ reported that cattle were kept until eight years old (or longer if reproductively proficient) therefore we believe that an average in-herd residency of 10 years is an appropriate approximation.

**Table 2:** Model parameters and the values used in the model.

Parameter	Value	Units	Range of values	References
R_0_	4			2–12 (modelling, Far North Cameroon)^24^ Infinity (final size calculation)^31^ 2.52 (final size calculation, vaccinated calf population)^32^ 14 (unpublished observation)^33^ 2.4–3.8 (estimated before an outbreak was officially reported (prior to mass vaccination), Argentina)^34^ 67 (58–78) and 88 (75–102) (naïve population, India) 8.04 (6.96–9.36) and 10.56 (9–12.24) (R_t_ vaccinated population, India)^35^
Herd size (N)	70			^27^
Transmission parameter (β)			Calculated for each scenario	Next-Generation matrix approach^64^
Mean duration of latent period $(\frac{1}{{\rm{\lambda }}})$	4.6	days		^36^ 3.1–4.8 days latent period^37^
Mean duration of infectious period $(\frac{1}{{{\rm{\sigma }}}_{I}})$	1.7	days		^36^
Probability of becoming a carrier (θ)	0		0.5	^38,39^
Ratio transmission from carrier: transmission from infectious individuals (ω)	0		0.000 to 0.024	$\frac{1}{500}$ estimated by Tenzin *et al*.^18^
Mean duration of the carrier state $(\frac{1}{{{\rm{\sigma }}}_{C}})$	182.625	days		^61,62^
Mean in-herd residency $(\frac{1}{{\rm{\mu }}})$	3652.5	days	365.25 days to 7305 days	11.08 years^65^ Fulani tend to keep their cattle until they are 8 years old (or longer if they are reproductively proficient)^51^

The duration of the carrier state (T_carrier_) is often reported to be 3.5 years^38,59,60^ but this likely represents the upper limit. Several studies have suggested shorter T_carrier_ with estimates ranging from 6 to 13 months^57,61,62^. Therefore, we have chosen to assume a relatively short T_carrier_ of 6 months (per-capita rate of recovery from the carrier state is $\frac{1}{6}$ months^−1^). Virus likely persists in the majority of individuals for several months after infection^63^ however estimates of the proportion affected varies. We have chosen to use a 50% probability of becoming a carrier (θ) in our model. This is consistent with values reported by Moonen and Schrijver and Arnold *et al*.^38,39^. We do not know if carriers can transmit FMD, but if possible, it is highly likely that the transmission rate from carriers will be low compared to that from infectious individuals. One study has estimated the relative transmission rate from carriers (ω) as $\frac{1}{500}=0.002$ that from infection individuals^18^. We have therefore chosen to vary ω between 0.000 and 0.025 allowing us to explore the impact of carriers on dynamics of FMD and persistence.

### Modelled scenarios

We have used two scenarios to answer questions about FMD persistence and within-herd dynamics posed in the Introduction. Scenario A explored the dynamics during and after a single outbreak. Outbreaks were initiated with a single infectious individual. The remainder of the population were either susceptible or recovered. Simulations were initiated with 50, 75 or 100% of the herd being susceptible. This reflects the range of within-herd sero-prevalence in endemic regions^45^. Scenario A was used to explore: disease persistence within a single herd; the period of reduced risk (T_ReducedRisk_) following a large outbreak and how this can be affected by herd management. Scenario B simulated two outbreaks. The first of these outbreaks was initiated in the same way as scenario A. The second outbreak occurred a set time after the end of the previous outbreak. The second outbreak was initiated by a susceptible individual becoming exposed (S to E) representing exposure from sources outwith the herd. Set inter-outbreak periods of between 1 and 30 years were used when exploring how memory of a large outbreak affects the future risk of an outbreak in a herd. The short inter-outbreak period of 1.2 years was used to explore disease burden as an approximate reflection of the frequency of outbreaks observed in the field^7,44^. We modelled these scenarios with and without a carrier state collecting information from the simulations to investigate how carriers alter dynamics at the herd-level.

### Model statistics

The following statistics were calculated from simulation results: outbreak size and duration; disease and viral persistence; T_ReducedRisk_; and disease burden. Outbreak size counts the number of individuals that become infectious during the outbreak. Outbreak duration (disease persistence) was defined as the time during which at least one exposed or infectious individual was present in the population and viral persistence as the time during which at least one exposed, infectious or carrier individual was present in the population. T_ReducedRisk_ was measured as the period of time from the end of an outbreak until the effective reproductive number R_t_ was greater than 1. Disease burden was calculated for infectious and recovered individuals as total number of individual-days spent within each state respectively. Infectious disease burden aims to account for the cost of FMD during an outbreak whilst the recovered disease burden is almost entirely made up of the effects of FMD felt after an outbreak has ended. Overall burden combined infectious and recovered burden weighting a recovered day as a proportion of an infectious day. Reported mean values are calculated from 10000 simulations.

**Figure 7:**
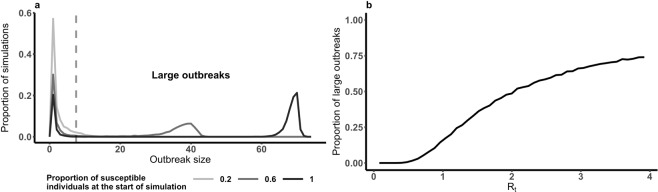
Higher herd immunity reduces probably of large outbreaks. There are fewer large outbreaks when herd immunity is higher (and the effective reproductive number (R_t_) is smaller) (**b**) and large outbreaks are smaller (**a**).

The distribution of outbreak size is affected by the proportion of susceptible individuals in the population prior to an outbreak (Fig. 7a). In a fully susceptible population outbreak size follows a bimodal distribution. Outbreaks simulated in a herd with 70 individuals infect between 1 and 7 individuals or 61 and 72 individuals. From this we have defined a large outbreak as any in which more than 10% of individuals become infectious. As herd susceptibility falls the size of large outbreaks gets smaller and there are fewer large outbreaks. Average outbreak size varies with the immunity in the herd prior to the outbreak (Fig. 7b). The basic reproduction number (R_0_), calculated for each model using the next generation matrix approach^64^ (${{R}_{0}}^{SEIR}=\frac{\beta \lambda }{(\lambda +\mu )({\sigma }_{I}+\mu )}$ and ${{R}_{0}}^{SEIRC}={{R}_{0}}^{SEIR}(1+\frac{\omega \theta {\sigma }_{I}}{({\sigma }_{C}+\mu )})$) refers to the average number of new cases resulting from a typically infectious individual in a fully susceptible population (see Supplementary Materials). Similarly R_t_ (${R}_{t}={R}_{0}(\frac{{S}_{(t)}}{{N}_{(t)}})$) is a measure of the average number of new cases resulting from a typically infectious individual in a partially susceptible population. Herds are considered to be at reduced risk of further outbreaks whilst R_t_ is less than 1. Figure 7b shows that high herd immunity (small R_t_) results in smaller outbreaks on average. We have utilised R_t_ to define the period of reduced risk following an outbreak.

## Supplementary information

Supplementary Material

## Data Availability

The code used to generate and analyse the data during the current study will be made available to readers upon publication.
