# Supplementary material for: Endemic foot and mouth disease: pastoral in-herd disease dynamics in sub-Saharan Africa
Source: Sci Rep. 2019 Nov 22;9:17349. doi: 10.1038/s41598-019-53658-5 (PMC6874544; doi:10.1038/s41598-019-53658-5)
Supplement: Supplementary file 1 — Supplementary Material [file 41598_2019_53658_MOESM1_ESM.pdf]

# Endemic foot and mouth disease: pastoral in-herd disease dynamics in sub-Saharan Africa

I. McLachlan[1,2*], G. Marion[2], I. J. McKendrick[2], T. Porphyre[1], I. G. Handel[1,3], B. M. deC. Bronsvoort[1]

[1]*The Epidemiology Economics and Risk Assessment (EERA) Group, The Roslin Institute and Royal (Dick) School of Veterinary Studies, University of Edinburgh, Easter Bush, Edinburgh, Midlothian, Scotland, United Kingdom*

[2]*Biomathematics and Statistics Scotland, Edinburgh, United Kingdom*

[3]*Royal (Dick) School of Veterinary Studies, University of Edinburgh, Easter Bush, Edinburgh, Midlothian, Scotland, United Kingdom*

*Corresponding author

Isobel.McLachlan@bioss.ac.uk

## Supplementary material

### Deterministic equations on which the stochastic model was based

$$\frac{dS}{dt} = births + \delta R - \frac{\beta S(I + \omega C)}{N} - \mu S$$

$$\frac{dE}{dt} = \frac{\beta S(I + \omega C)}{N} - \lambda E - \mu E$$

$$\frac{dI}{dt} = \lambda E - \sigma_I I - \mu I$$

$$\frac{dC}{dt} = \theta \sigma_I I - \sigma_C C - \mu C$$

$$\frac{dR}{dt} = (1 - \theta)\sigma_I I + \sigma_C C - \delta R - \mu R$$

### Next Generation Matrix Proof $R_0$ equation

$$\dot{S} = births + \delta R - \frac{\beta SI}{N} - \frac{\omega \beta SC}{N} - \mu S$$

$$\dot{E} = \frac{\beta SI}{N} + \frac{\omega \beta SC}{N} - \lambda E - \mu E$$

$$\dot{I} = \lambda E - \sigma_I I - \mu I$$

$$\dot{C} = \theta \sigma_I I - \sigma_C C - \mu C$$

$$\dot{R} = (1 - \theta)\sigma_I I + \sigma_C C - \delta R - \mu R$$

Linearise (S=N, I=0, C=0, R=0) – disease free equilibrium

$$\dot{E} = \beta I + \omega \beta C - (\lambda + \mu)E$$

$$\dot{I} = \lambda E - (\sigma_I + \mu)I$$

$$\dot{C} = \theta \sigma_I I - (\sigma_C + \mu)C$$

Disease states

$$y = \begin{pmatrix} E \\ I \\ C \end{pmatrix}$$

$$\dot{y} = \begin{pmatrix} \dot{E} \\ \dot{I} \\ \dot{C} \end{pmatrix} = Ay$$

$$\dot{y} = \begin{pmatrix} A_{EE} & A_{EI} & A_{EC} \\ A_{IE} & A_{II} & A_{IC} \\ A_{CE} & A_{CI} & A_{CC} \end{pmatrix} \begin{pmatrix} E \\ I \\ C \end{pmatrix}$$

$$= \begin{pmatrix} A_{EE}E & +A_{EI}I & +A_{EC}C \\ A_{IE}E & +A_{II}I & +A_{IC}C \\ A_{CE}E & +A_{CI}I & +A_{CC}C \end{pmatrix} = \begin{pmatrix} -(\lambda+\mu)E & +\beta I & +\omega\beta C \\ \lambda & -(\sigma_I+\mu) & +0 \\ 0 & +\theta\sigma_I I & -(\sigma_C+\mu) \end{pmatrix}$$

$$A = \begin{pmatrix} -(\lambda+\mu) & +\beta & +\omega\beta \\ \lambda & -(\sigma_I+\mu) & +0 \\ 0 & +\theta\sigma_I & -(\sigma_C+\mu) \end{pmatrix}$$

Decompose $A = T + \sum$

$$T = \begin{pmatrix} 0 & \beta & \omega\beta \\ 0 & 0 & 0 \\ 0 & 0 & 0 \end{pmatrix}$$

$$\sum = \begin{pmatrix} -(\lambda+\mu) & 0 & 0 \\ \lambda & -(\sigma_I+\mu) & 0 \\ 0 & \theta\sigma_I & -(\sigma_C+\mu) \end{pmatrix}$$

Using the Next-Generation matrix (NGM) approach by Diekmann *et al* [64]

$$NGM = K = -T{\textstyle\sum}^{-1}$$

$$-{\textstyle\sum}^{-1} = \begin{pmatrix} \frac{1}{(\lambda+\mu)}, & 0, & 0 \\ \frac{\lambda}{(\lambda+\mu)(\sigma_I+\mu)}, & \frac{1}{(\sigma_I+\mu)}, & 0 \\ \frac{\lambda\theta\sigma_I}{(\lambda+\mu)(\sigma_I+\mu)(\sigma_C+\mu)} & \frac{\theta\sigma_I}{(\sigma_C+\mu)(\sigma_I+\mu)} & \frac{1}{(\sigma_C+\mu)} \end{pmatrix}$$

$$K = -T{\textstyle\sum}^{-1} = \begin{pmatrix} K_{EE} & K_{EI} & K_{EC} \\ 0 & 0 & 0 \\ 0 & 0 & 0 \end{pmatrix}$$

$$K_{EE} = \frac{\beta\lambda}{(\lambda+\mu)(\sigma_I+\mu)} + \frac{\omega\beta\lambda\theta\sigma_I}{(\lambda+\mu)(\sigma_I+\mu)(\sigma_C+\mu)}$$

The eigenvalues of K are the solutions to

$$(K_{EE} - \phi)\phi^2 = 0$$

$$\phi = 0, \phi = 0, \phi = K_{EE}$$

$R_0$ is the largest eigenvalue of the NGM K

$$R_0 = K_{EE}$$

$$= \frac{\beta\lambda}{(\lambda+\mu)(\sigma_I+\mu)} + \frac{\omega\theta\sigma_I}{(\sigma_C+\mu)} \times \frac{\beta\lambda}{(\lambda+\mu)(\sigma_I+\mu)}$$

$$= \frac{\beta\lambda}{(\lambda+\mu)(\sigma_I+\mu)}\left(1 + \frac{\omega\theta\sigma_I}{(\sigma_C+\mu)}\right)$$

If $\omega = 0$ or $\theta = 0$ or $\sigma_C \to \infty$ the model collapses to the SEIR model – therefore

$$R_0{}^{SEIR} = \frac{\beta\lambda}{(\lambda + \mu)(\sigma_I + \mu)}$$

$$R_0{}^{SEIRC} = R_0{}^{SEIR}\left(1 + \frac{\omega\theta\sigma_I}{(\sigma_C + \mu)}\right)$$

## Additional figures

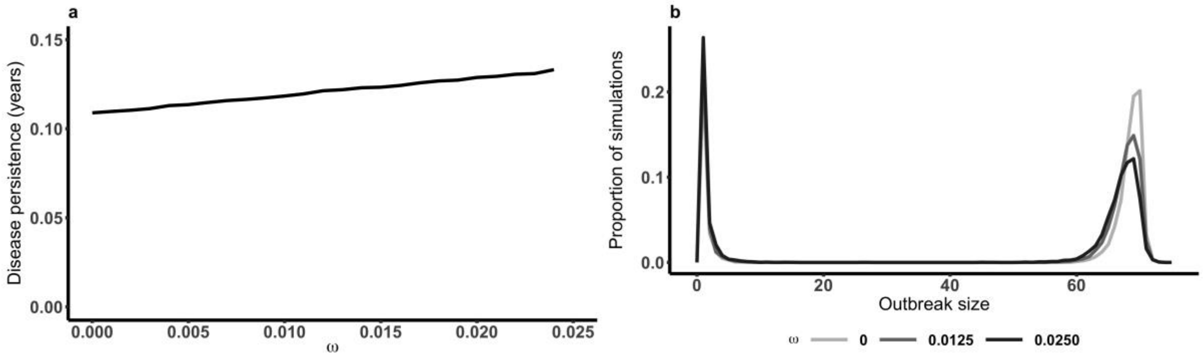


*Figure S1 – **Additional carrier graphs.** Disease persistence (or outbreak duration) increases as relative transmission from carriers ($\omega$) increases (a). There are fewer large outbreaks when $\omega$ is higher (b).*

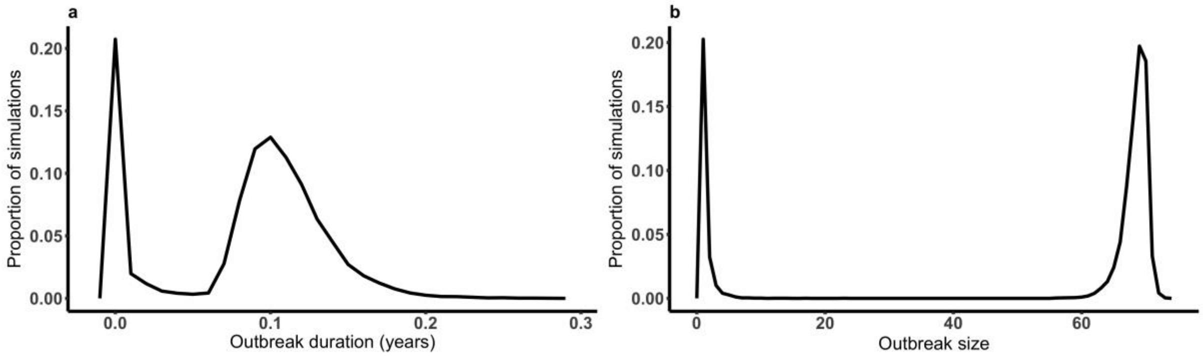


*Figure S2 – **Disease cannot persist within a single herd with virus transmitting carriers.** The mean duration of large outbreaks is 0.084 years (1.0 month) with 100% of outbreaks lasting less than 0.28 years (3.3 months) (a). The majority of outbreaks are large although some small outbreaks are observed (b). In the results shown here relative transmission from carriers is 0.002 that of transmission from infectious individuals.*

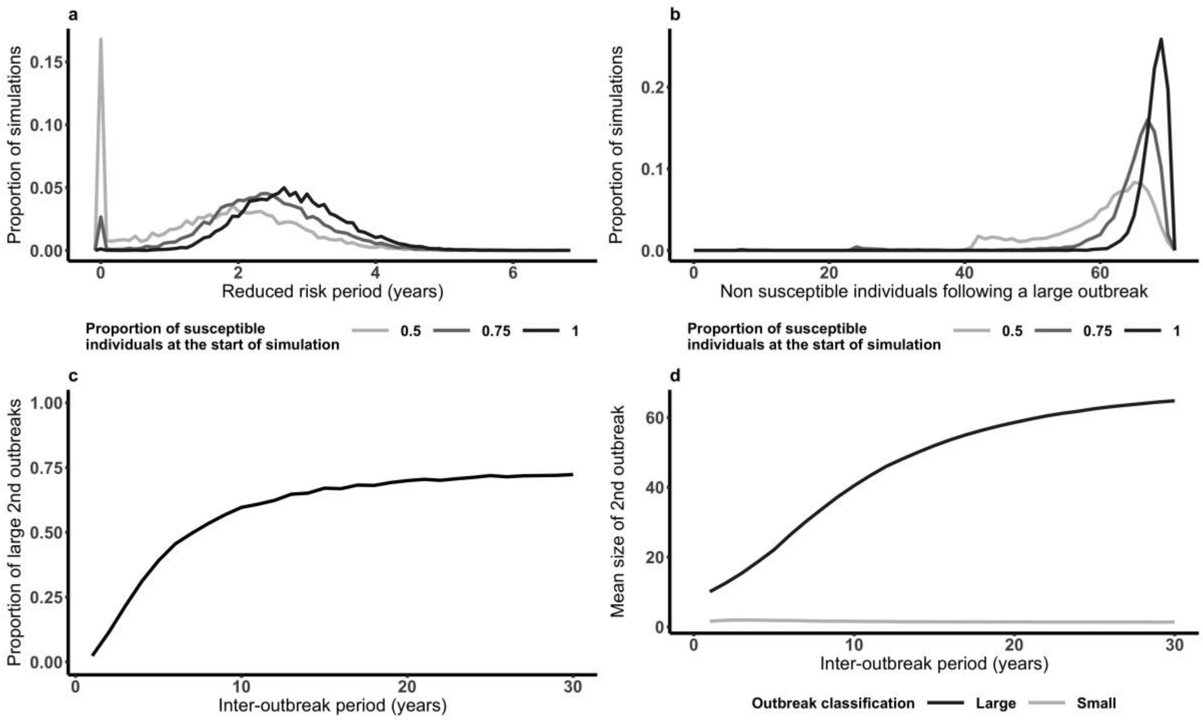


*Figure S3 –* ***Memory of herd immunity following a large outbreak.*** *When relative transmission from carriers is 0.002 times that from infectious individuals the trends observed are similar to when no carriers are present. Following an outbreak there is a period where the herd is at reduced risk of further outbreaks (a). Immunity within the herd is higher when susceptibility prior to the outbreak is higher (b). The longer the interval between outbreaks the greater the probability that the subsequent outbreak will be large (c) and these large outbreaks will infect more individuals (d).*

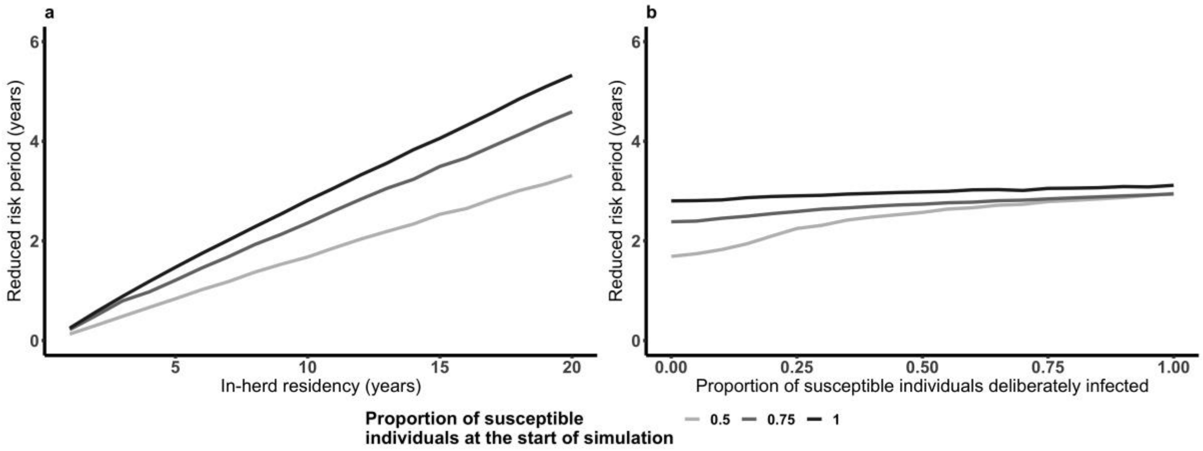


*Figure S4 –* ***Impact of herd management on the reduced risk period ($T_{ReducedRisk}$) following a large outbreak.*** *Transmission from carriers is 0.002 relative to transmission from infectious individuals. Shorted average in-herd residency shortens $T_{ReducedRisk}$ (a – the rate of population turn-over ($\mu$) is varied), increasing the proportion of individuals deliberate infected results in a longer mean $T_{ReducedRisk}$ following an outbreak (b – once an outbreak is detected deliberate infection of a*

*proportion of the remaining susceptible individuals occurs).*

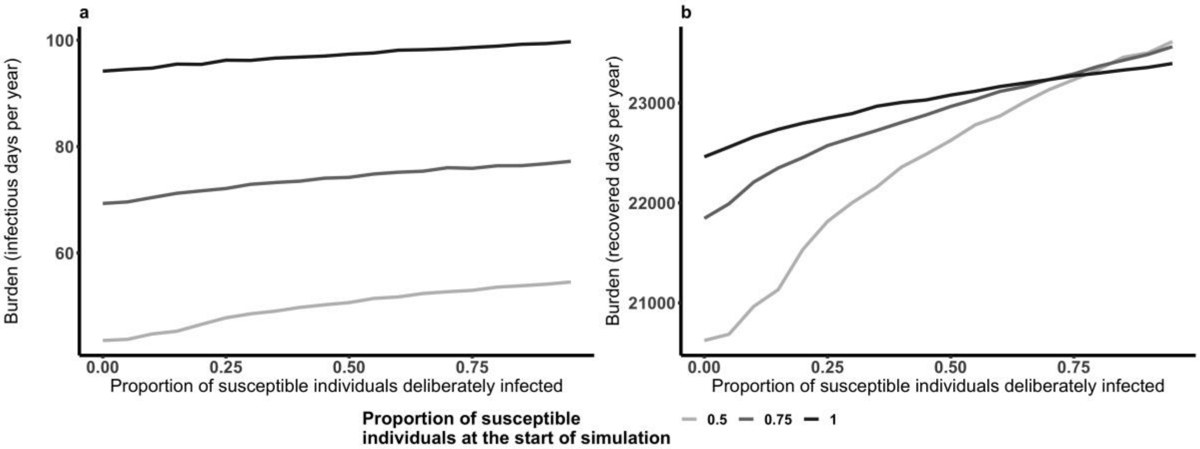


*Figure S5 – **Deliberate infection increases disease burden.** Increased the proportion of the susceptible population deliberately infected following outbreak detection increases the mean disease burden associated with both infectious individuals (a) and recovered individuals (b). SEIR model (no carriers).*

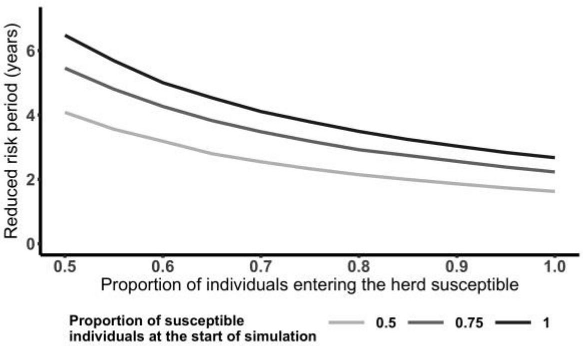


*Figure S6 – **The mean reduced risk period is shorter when a greater proportion of replacement individuals are susceptible**.*
